# Supplementary material for: Cold plasma-induced transcriptomic reprogramming and alternative splicing in tomato plants infected with ToBRFV
Source: PLoS One. 2026 Jan 5;21(1):e0338036. doi: 10.1371/journal.pone.0338036 (PMC12768285; doi:10.1371/journal.pone.0338036)
Supplement: S2 Table — (PDF) [file pone.0338036.s002.pdf]

Supplementary Table 2. Predicted interactions between tomato miRNAs and the alternative splicing gene under cold plasma treatment	
miRNA_Acc.	Target_Acc.
sly-miR10532	Solyc12g056650.2
sly-miR399	Solyc09g082060.3
sly-miR7981e	Solyc12g056650.2
sly-miR7981f	Solyc12g056650.2
sly-miR7981f	Solyc01g060470.3
sly-miR10541	Solyc06g066060.3
sly-miR159b	Solyc04g082400.3
sly-miR390a-5p	Solyc09g072900.3
sly-miR390b-5p	Solyc09g072900.3
sly-miR403-5p	Solyc08g066800.3
sly-miR477-3p	Solyc11g066620.2
sly-miR5302b-3p	Solyc01g100050.3
sly-miR7981c	Solyc03g096950.3
sly-miR7981d	Solyc03g096950.3
sly-miR10532	Solyc01g060470.3
sly-miR10541	CCD1B
sly-miR156e-3p	Solyc09g065540.3
sly-miR156e-3p	Solyc12g056650.2
sly-miR159	Solyc01g057770.3
sly-miR164a-5p	Solyc12g056650.2
sly-miR164b-5p	Solyc12g056650.2
sly-miR168b-3p	Solyc02g069720.3
sly-miR171c	Solyc10g054330.2
sly-miR171d	Solyc07g008950.3
sly-miR171e	CCD1B
sly-miR172d	Solyc03g115247.1
sly-miR1918	Solyc04g015270.3
sly-miR1919a	Solyc01g060470.3
sly-miR1919b	Solyc01g060470.3
sly-miR1919c-3p	Solyc01g060470.3
sly-miR391	Solyc09g082060.3
sly-miR394-3p	Solyc04g082400.3
sly-miR394-3p	Solyc10g074980.2
sly-miR395a	Solyc09g098240.3
sly-miR395a	Solyc04g015270.3
sly-miR395b	Solyc09g098240.3
sly-miR395b	Solyc04g015270.3
sly-miR403-5p	Solyc02g030250.3
sly-miR403-5p	Solyc01g091770.3

sly-miR482c	Solyc10g074980.2
sly-miR482d-3p	Solyc09g014620.3
sly-miR530	Solyc11g066610.2
sly-miR5302b-3p	Solyc01g100050.3
sly-miR5303	Solyc09g098240.3
sly-miR5303	Solyc09g098240.3
sly-miR6022	Solyc09g098240.3
sly-miR6023	Solyc10g054330.2
sly-miR6024	Solyc11g066610.2
sly-miR7981e	Solyc01g060470.3
sly-miR7981f	Solyc04g082400.3
sly-miR9475-5p	Solyc12g021280.2
sly-miR9477-5p	Solyc03g117950.3
sly-miR9479-5p	Solyc02g014150.3
sly-miR10533	Solyc05g051790.3
sly-miR10533	Solyc09g061840.3
sly-miR10535b	Solyc07g006620.3
sly-miR10539	Solyc10g074980.2
sly-miR10540	COP1
sly-miR10541	CB5-A
sly-miR156a	Solyc02g071280.3
sly-miR156b	Solyc02g071280.3
sly-miR156c	Solyc02g071280.3
sly-miR156d-3p	Solyc09g098240.3
sly-miR156e-3p	Solyc09g025250.3
sly-miR159	Solyc01g100050.3
sly-miR166a	Solyc02g014150.3
sly-miR166b	Solyc02g014150.3
sly-miR166c-3p	Solyc02g014150.3
sly-miR167b-3p	Solyc03g043760.3
sly-miR168a-3p	Solyc02g069720.3
sly-miR169e-3p	Solyc02g071280.3
sly-miR171b-3p	Solyc07g008950.3
sly-miR171c	Solyc01g100050.3
sly-miR172a	Solyc09g098240.3
sly-miR172b	Solyc09g098240.3
sly-miR1916	Solyc07g063870.3
sly-miR1918	Solyc04g054190.3
sly-miR1918	Solyc03g117950.3
sly-miR319a	Solyc12g021280.2
sly-miR319a	dxs2

sly-miR319a	Solyc01g057770.3
sly-miR319a	Solyc09g025250.3
sly-miR319b	Solyc12g021280.2
sly-miR319b	dxs2
sly-miR319b	Solyc01g057770.3
sly-miR319c-3p	Solyc12g021280.2
sly-miR319c-3p	Solyc01g057770.3
sly-miR319c-3p	dxs2
sly-miR319c-5p	Solyc05g007500.3
sly-miR390a-5p	Solyc01g060470.3
sly-miR390a-5p	Solyc03g096950.3
sly-miR391	Solyc08g081300.3
sly-miR393	CCD1B
sly-miR394-3p	Solyc09g098240.3
sly-miR395a	Solyc07g043390.3
sly-miR395a	Solyc12g056650.2
sly-miR395b	Solyc07g043390.3
sly-miR395b	Solyc12g056650.2
sly-miR396a-5p	Solyc04g054190.3
sly-miR396a-5p	Solyc01g100050.3
sly-miR396b	Solyc04g054190.3
sly-miR396b	Solyc01g100050.3
sly-miR397-5p	Solyc08g066800.3
sly-miR403-3p	Solyc03g117950.3
sly-miR482a	Solyc10g054330.2
sly-miR482e-5p	Solyc01g100050.3
sly-miR530	Solyc05g007500.3
sly-miR5302a	Solyc09g098240.3
sly-miR5302b-3p	Solyc03g119140.3
sly-miR5303	Solyc09g065540.3
sly-miR6022	Solyc03g096950.3
sly-miR6023	Solyc02g071280.3
sly-miR6023	Solyc03g117950.3
sly-miR6023	Solyc07g006620.3
sly-miR6024	Solyc09g098240.3
sly-miR6025	Solyc12g056650.2
sly-miR827	Solyc03g117950.3
sly-miR9469-5p	Solyc09g011160.3
sly-miR9469-5p	Solyc02g071280.3
sly-miR9472-3p	Solyc07g062080.3
sly-miR9472-5p	Solyc04g082400.3

sly-miR9473-5p	Solyc01g057770.3
sly-miR9475-3p	Solyc04g082400.3
sly-miR9477-5p	Solyc12g021280.2
