# Supplementary material for: Cold plasma-induced transcriptomic reprogramming and alternative splicing in tomato plants infected with ToBRFV
Source: PLoS One. 2026 Jan 5;21(1):e0338036. doi: 10.1371/journal.pone.0338036 (PMC12768285; doi:10.1371/journal.pone.0338036)
Supplement: Attachment — Submitted filename: comments.docx [file pone.0338036.s003.docx]

Comments :

Praised the innovative application of CAP to address a critical agricultural problem and its focus on a nuanced molecular mechanism, as highlighted in the abstract

1. The abstract highlights significant AS changes, predominantly exon skipping and intron retention, linked to disease resistance chromosomes, further underscoring the innovative approach of the study in identifying specific molecular responses.
2. While the introduction presents plasma as a novel antiviral tool, it lacks a clear gap statement comparing this work to existing transcriptomic or AS-based studies in plasma-treated plants. The introduction could briefly explain the mechanistic link between plasma-induced reactive species and transcriptional/splicing regulation in plants. This would strengthen the biological plausibility of the findings.
3. Including more replicates provides strength in statistical detection of RNA-seq and AS detection
4. All raw RNA-seq reads have been deposited in the NCBI Sequence Read Archive (SRA) under accession number [to be added upon acceptance]. The Python scripts and CLC pipelines used for AS filtering, FASTA extraction, and KEGG annotation are publicly available on GitHub ([insert link]) and archived via Zenodo for reproducibility.
5. Only splicing junctions supported by a minimum of 10 uniquely mapped reads and detected in at least one replicate were considered high-confidence AS events. Annotation was based on the Solanum lycopersicum reference genome SL4.0 and ITAG v4.1 annotation.
6. Principal component analysis (PCA) and hierarchical clustering (Supplementary Fig. S1) showed distinct expression profiles between plasma-treated and control samples, confirming robust transcriptome separation.
7. Clearly describe the specific statistical method used to identify differential alternative splicing (DAS) events between the plasma-treated and control conditions. For instance, specify if a dedicated tool like rMATS, SUPPA2, dsplice, or ASprofile was employed, along with the exact false discovery rate (FDR)-adjusted p-value threshold (e.g., FDR < 0.05) and delta PSI (ΔΨ) cutoffs (e.g., |ΔΨ| > 0.1) for classifying significant AS events. This is crucial for establishing analytical rigor and reproducibility.
8. Provide a comprehensive, step-by-step RT-PCR protocol for ToBRFV validation. This must include the exact sequences of the forward and reverse primers used, the specific RNA extraction kit or method for validation samples (if not identical to the RNA-Seq protocol), and all reaction conditions (e.g., cDNA synthesis parameters, PCR amplification cycles, annealing temperatures). This level of detail is essential for transparency and replication.
9. The description of AS event types in identifies exon skipping (ES), intron retention (IR), and mutually exclusive exons (MXE) were the most common. However, the paper fails to quantify the relative proportions or exact frequencies of each AS type. This omission makes it challenging to assess the dominant AS mechanisms at play and to rigorously compare these findings with established patterns in plant AS, where intron retention is typically the most prevalent form. This impacts the analytical validity and interpretability of the AS landscape
10. The discussion in regarding AS events on chromosome 9 (related to Tm-2 gene) and chromosome 3 (resistance locus) is highly speculative. While these are indeed relevant genomic regions for resistance, the paper does not present direct empirical evidence linking the identified AS events to these specific genes or loci. The mere co-localization of AS events with known resistance genes on a chromosome is insufficient to imply a functional relationship, thus severely impacting the analytical validity and interpretability of these claims.
11. The integration of AS and DEG analyses in identifies '19 such genes'. However, Table 1 lists genes explicitly marked 'C' (control) which implies unique AS events specific to the control condition, directly contradicting the stated criterion for DE-ASGs in that requires 'presence of unique AS events specific to the plasma-treated condition'. This inconsistency between methodology definition and reported results critically undermines the analytical validity and transparency of the DE-ASG classification.
12. Out of 349 AS events, 272 were mapped to 214 unique genes, confirming that multiple splicing types can occur within the same locus.
13. The Methods section lacks critical details required for reproducibility. Key omissions include the specific model and configuration of the plasma device, the precise timing of the plasma treatment relative to infection and seedling growth, and the number of individual plants pooled for each biological replicate.
14. The statement that "ToBRFV surpasses resistance conferred by tomato and pepper R genes (Tm-1, Tm-2, Tm-22, L1, L2), and no resistant tomato cultivars are commercially available" highlights a critical research gap, but the paper does not adequately review the latest developments in ToBRFV resistance breeding or recent attempts to overcome this challenge, which would further position the novelty of the plasma treatment as an alternative strategy.
15. The paper generally describes alternative splicing (AS) in plants and its role in plant-virus interactions. However, it does not sufficiently acknowledge the growing body of literature on how viruses modulate host AS or how AS plays a role in viral infection processes. This omission weakens the argument for the novelty of investigating AS in virus-infected plants under plasma treatment, as it implies a lack of awareness of this existing research domain.
16. The paper generally describes alternative splicing (AS) in plants and its role in plant-virus interactions. However, it does not sufficiently acknowledge the growing body of literature on how viruses modulate host AS or how AS plays a role in viral infection processes. This omission weakens the argument for the novelty of investigating AS in virus-infected plants under plasma treatment, as it implies a lack of awareness of this existing research domain.
17. The logical jump in from AS to 'improved defense-related gene expression' and 'priming plants' lacks mechanistic clarity. It doesn't explain how specific AS isoforms contribute to this improvement or what specific molecular events constitute 'priming' in this context, presenting a plausible hypothesis as a direct consequence without detailed support, which is a logical gap.
18. about resistance genes (Tm-2, Tm-1) on chromosomes 9 and 3, and the observed 'increase in AS events,' presents a logical gap. It does not clearly articulate whether these AS events occur within these resistance genes, in nearby regulatory elements, or in other genes on those chromosomes, leaving the specific connection to resistance mechanisms unclear.
19. The discussion on the ubiquitin-mediated proteolysis pathway in presents a logical gap by not clearly establishing how alternative splicing events are connected to it. While stating that AS 'plays a central role' and 'splicing variations' occur, it doesn't specify the direct causal or correlational link for optimal clarity and coherence.
20. In the statement 'The miR395a/b regulates networks that could modulate stress responses' is vague and lacks specificity regarding the mechanism of modulation. Using 'could' rather than a more definitive statement weakens the clarity of the claims about miRNA function.
