# Supplementary material for: Cold plasma-induced transcriptomic reprogramming and alternative splicing in tomato plants infected with ToBRFV
Source: PLoS One. 2026 Jan 5;21(1):e0338036. doi: 10.1371/journal.pone.0338036 (PMC12768285; doi:10.1371/journal.pone.0338036)
Supplement: Attachment — Submitted filename: Response to reviwers.docx [file pone.0338036.s005.docx]

Dear Editor,

We are pleased to submit the revised version of our manuscript entitled “Cold Plasma-Induced Transcriptomic Reprogramming and Alternative Splicing in Tomato Plants Infected with ToBRFV” (Manuscript ID: PONE-D-25-42103) for consideration in PLOS ONE.

We sincerely thank you and the reviewers for your thoughtful and constructive feedback, which has significantly helped us improve the quality and clarity of our work. In response to the reviewers’ comments, we have carefully revised both the manuscript and the supplementary materials. All responses to the reviewers’ concerns are provided in the attached “Response to Reviewers” document, where:

- The reviewers’ original comments are presented in red,
- Our point-by-point replies are provided directly beneath each comment,
- All modifications made to the manuscript are clearly indicated using track-changes in the revised manuscript file.

We believe the revised manuscript now presents a more robust, transparent, and well-contextualized study that aligns with the high standards of PLOS ONE. We appreciate your time and consideration and look forward to your decision.

Sincerely,

Mahsa Rostami

On behalf of all co-authors

Reviewer #1: Dear Author,

This study, titled "Cold Plasma-Induced Transcriptomic Reprogramming and Alternative Splicing in Tomato Plants Infected with ToBRFV," investigates the use of cold atmospheric plasma (CAP), specifically cold air glow discharge plasma (CAGDP), as an innovative approach to maintain plant defenses against the Tomato Brown Rugose Fruit Virus (ToBRFV) in tomato seedlings. The manuscript is clearly written and well-organized. However, it is primarily an in silico study, utilizing many bioinformatic tools. Although it offers novel insights into the effects of cold atmospheric plasma (CAP) on alternative splicing (AS):

**Q1:**The study was performed on only one tomato cultivar (SV3725TH) and at a single time point (four-leaf stage seedlings) after treatment.

**Response:** We sincerely appreciate the reviewer’s insightful comment. The reviewer is absolutely correct that our current study focused on a single commercial tomato cultivar (SV3725TH) and a single developmental time point (four-leaf stage) post cold plasma treatment. This experimental design was chosen based on our prior work , which demonstrated that 3-minute CAGDP treatment of ToBRFV-infected seeds of SV3725TH significantly reduced viral accumulation and enhanced seedling vigor specifically at the four-leaf stage, making it the optimal window to capture early molecular responses linked to antiviral defense. That said, we fully acknowledge that temporal dynamics and genetic background can significantly influence alternative splicing and transcriptomic reprogramming. As highlighted in the “Limitations and Future Directions” section, we recognize this as a limitation and explicitly propose that future studies should expand to multiple time points and diverse cultivars—including resistant and susceptible genotypes—to assess the robustness and generalizability of plasma-induced splicing responses. To address this concern transparently, we have now strengthened the discussion of this limitation in the revised manuscript and emphasized that while our findings provide a foundational molecular map of CAGDP action in a commercially relevant context, broader validation across genotypes and time courses will be essential for translational applications.

These sentences were added to the main text:

"While this study provides novel insights into the transcriptomic and splicing responses of tomato to cold plasma under viral stress, several limitations warrant further investigation. First, the analysis was restricted to a single susceptible cultivar (SV3725TH) and a single post-treatment time point (four-leaf stage). Although this stage was selected based on prior evidence of maximal antiviral effect and physiological response to CAGDP (3), it may not capture the full dynamics of splicing regulation over time. Future work should include longitudinal sampling and a panel of cultivars with varying genetic backgrounds—including those carrying known resistance genes (e.g., Tm-1, Tm-2²)—to determine whether the observed AS patterns are conserved or genotype-specific."

**Q2:** The functional roles of the identified alternatively spliced genes and their corresponding isoforms still need to be validated through wet lab experiments (main drawbacks).

**Response:** We sincerely thank the reviewer for this important observation. The reviewer is absolutely correct that the functional validation of the identified alternatively spliced (AS) genes and their protein isoforms remains a key limitation of our current study, which is primarily based on RNA-Seq and computational prediction.

Our work was designed as a discovery-phase, hypothesis-generating investigation to map the transcriptomic and splicing landscape induced by cold plasma in ToBRFV-infected tomato seedlings. The identification of 74 plasma-specific AS events—including exon skipping and intron retention in resistance-associated chromosomes—and the integration with differential expression and miRNA targeting provide a strong foundation for future functional studies.

We fully agree that wet-lab validation is essential to confirm:

(i) whether the predicted splice variants are indeed translated into stable protein isoforms,

(ii) how these isoforms affect subcellular localization, protein–protein interactions, or enzymatic activity, and

(iii) their direct contribution to antiviral defense.

To address this concern transparently, we have expanded the “Limitations and Future Directions” section to explicitly state that functional characterization via techniques such as RT-PCR with isoform-specific primers, Western blotting (if antibodies are available), transient overexpression, CRISPR/Cas9-mediated isoform knockout, or virus-induced gene silencing (VIGS) will be critical next steps. We are already planning such experiments for key candidates like Solyc07g043390.3 (glycosyltransferase) and Solyc09g011160.3, which showed both plasma-specific splicing and significant upregulation.

We appreciate the reviewer’s emphasis on this point, as it strengthens the translational relevance of our findings. While beyond the scope of this initial omics study, we now clearly frame these validations as essential future work to move from correlation to causation in plasma-induced antiviral mechanisms.

These sentences were added to the main text:

" Another major limitation of the present study is the lack of experimental validation of the functional consequences of the identified alternative splicing events. While our bioinformatic pipeline robustly detected plasma-specific isoforms and integrated them with expression and regulatory data, it remains unknown whether these splice variants produce stable proteins or alter protein function, localization, or interaction networks. Future studies should employ isoform-specific RT-PCR, proteomics, and targeted genetic approaches (e.g., CRISPR/Cas9 or VIGS) to validate the biological roles of key AS genes in mediating cold plasma-enhanced resistance to ToBRFV. Such functional validation will be crucial to translate these molecular insights into practical crop protection strategies."

**Q3:** Although miRNA-mRNA interactions were predicted bioinformatically using psRNATarget, an in vivo validation remains necessary.

**Response:** We sincerely thank the reviewer for this valuable and well-founded comment. The reviewer is absolutely correct that bioinformatic prediction alone cannot confirm functional miRNA–mRNA interactions, and in vivo or in planta validation is essential to substantiate the regulatory relationships proposed in our network (Fig. 6). In our study, we used psRNATarget with stringent criteria (expectation score < 3, based on complementarity, target accessibility, and evolutionary conservation) to generate high-confidence hypotheses about post-transcriptional regulation of plasma-induced alternatively spliced genes. This approach is widely accepted in initial discovery-phase studies, particularly when integrated with transcriptomic data, as it helps prioritize candidate regulatory pairs for downstream validation. However, we fully acknowledge that true validation requires experimental evidence, such as:

Degradome sequencing (PARE or GMUCT) to detect miRNA-guided cleavage fragments,

5′ RACE to confirm cleavage sites at predicted target positions,

Transient co-expression assays (e.g., in Nicotiana benthamiana) using miRNA mimics and reporter constructs fused to target gene 3′UTRs,

Or qRT-PCR correlation analysis between miRNA and target mRNA levels across time courses or treatments.

To address this concern transparently, we have revised the “Limitations and Future Directions” section to explicitly state that while our miRNA–mRNA network provides a testable framework for plasma-mediated post-transcriptional regulation, functional validation through degradome analysis or reporter assays will be critical in future work.

These sentences were added to the main text:

"While the miRNA–mRNA interaction network was constructed using stringent bioinformatic criteria, these predictions remain hypothetical without experimental confirmation. Future studies should employ degradome sequencing (e.g., PARE) to validate cleavage events, and transient expression assays to test the repressive effects of key miRNAs on their predicted alternatively spliced targets. Such validation will be essential to establish causal links between plasma-induced miRNA expression, AS regulation, and antiviral phenotypes. "

**Q4:** The study indicates that combining proteomics and metabolomics in future research could reveal the downstream effects of plasma-induced transcriptomic reprogramming and offer a more comprehensive understanding of plant-virus-plasma interactions. This shows that the current conclusion is incomplete. Minor typing errors, such as the "reference" (line 400), should be corrected. Additionally, the quality of the figures needs enhancement.

**Response:** We sincerely thank the reviewer for these thoughtful and constructive comments.

Regarding the completeness of the conclusions:

We agree that our current conclusions are based solely on transcriptomic and bioinformatic evidence and do not capture the full biological cascade—from RNA to functional proteins and metabolites. However, we intentionally framed our conclusions as a foundational, hypothesis-generating study that maps the molecular landscape of cold plasma action at the RNA level. The statement about integrating proteomics and metabolomics was included not to highlight incompleteness, but to acknowledge the natural progression of systems biology research and to guide future work. In the revised manuscript, we have refined the wording in the Conclusion and Limitations sections to clarify that our findings represent an initial molecular framework, and that multi-omics validation will be essential to establish causal links between splicing changes and physiological outcomes.

Regarding typographical errors:

We appreciate the reviewer’s careful reading. The word “reference” appearing inappropriately (e.g., on or near line 400) was an artifact of manuscript formatting during conversion.

Regarding figure quality:

In the manuscript, we have generated all figures at high resolution (minimum 300 dpi) in TIFF format.

These sentences were added to the main text:

" While the miRNA–mRNA interaction network was constructed using stringent bioinformatic criteria, these predictions remain hypothetical without experimental confirmation. Future studies should employ degradome sequencing (e.g., PARE) to validate cleavage events, and transient expression assays to test the repressive effects of key miRNAs on their predicted alternatively spliced targets. Such validation will be essential to establish causal links between plasma-induced miRNA expression, AS regulation, and antiviral phenotypes. Furthermore, our findings provide compelling evidence that CAGDP can reprogram transcriptomic and post-transcriptional networks in tomato under viral stress, offering a sustainable strategy to enhance crop resilience. While this RNA-level analysis establishes a foundational molecular framework, future integration of proteomics, metabolomics, and functional validation will be essential to fully elucidate the causal links between plasma-induced splicing changes and antiviral phenotypes."

Reviewer #2: Praised the innovative application of CAP to address a critical agricultural problem and its focus on a nuanced molecular mechanism, as highlighted in the abstract

Q1. The abstract highlights significant AS changes, predominantly exon skipping and intron retention, linked to disease resistance chromosomes, further underscoring the innovative approach of the study in identifying specific molecular responses.

**Response:** We sincerely thank the reviewer for this positive and insightful remark. We agree that the identification of plasma-induced alternative splicing events—particularly exon skipping and intron retention on chromosomes harboring known disease resistance loci (e.g., chromosomes 1, 3, and 9)—represents a key novelty of our work. These findings provide a molecular basis for the observed antiviral effects of cold plasma and open new avenues for understanding post-transcriptional regulation in plant defense. We have retained this emphasis in the revised manuscript

Q2. While the introduction presents plasma as a novel antiviral tool, it lacks a clear gap statement comparing this work to existing transcriptomic or AS-based studies in plasma-treated plants. The introduction could briefly explain the mechanistic link between plasma-induced reactive species and transcriptional/splicing regulation in plants. This would strengthen the biological plausibility of the findings.

**Response:** We sincerely thank the reviewer for this insightful suggestion. We agree that clarifying the research gap and providing a mechanistic rationale for plasma-induced transcriptional and splicing regulation would strengthen the biological context of our study. In response, we have revised the Introduction to explicitly state that, while cold plasma has been shown to reduce viral load and enhance plant growth, no prior study has investigated its impact on genome-wide alternative splicing patterns in virus-infected plants—particularly in the context of ToBRFV, a rapidly emerging pathogen that overcomes conventional resistance mechanisms.

These sentences were added to the main text (introduction):

" Although cold plasma has demonstrated efficacy in deactivating viruses and promoting plant vigor, its role in shaping the post-transcriptional landscape—particularly alternative splicing—remains unexplored in plant–virus systems. Notably, no transcriptomic study to date has examined how plasma-generated reactive oxygen species (ROS) influence splicing decisions in crops under viral stress. Emerging evidence suggests that ROS act as signaling molecules that can modulate the activity of splicing regulators through redox-sensitive cysteine residues, phosphorylation cascades, or chromatin remodeling, thereby altering splice site selection and isoform diversity (6). Given that AS is a rapid and reversible layer of gene regulation critical for immune responses, it is biologically plausible that CAGDP-induced ROS reprogram splicing networks to enhance antiviral defense— a hypothesis directly addressed in this study."

Q3. Including more replicates provides strength in statistical detection of RNA-seq and AS detection

**Response:** We sincerely thank the reviewer for this important observation. We fully agree that increasing the number of biological replicates would enhance the statistical robustness of RNA-Seq and alternative splicing analyses. In our study, we used two high-quality biological replicates per condition, which is consistent with several published plant RNA-Seq studies focusing on stress-induced splicing. Moreover, our sequencing depth was high (>35 million paired-end reads per sample, >93% mapping rate, Phred >30), which partially compensates for the limited number of replicates by improving detection sensitivity. That said, we acknowledge this as a limitation. We believe that despite this constraint, our key findings—such as plasma-specific AS enrichment on resistance-linked chromosomes (1, 3, 9), the identification of 19 DE-ASGs, and the integration with miRNA networks—are robust, reproducible across replicates, and supported by strong functional enrichment (FDR < 0.05). Nevertheless, we fully agree with the reviewer that future validation studies should include ≥3 biological replicates to strengthen statistical inference, and we will adopt this design in our follow-up experiments.

Q4. All raw RNA-seq reads have been deposited in the NCBI Sequence Read Archive (SRA) under accession number [to be added upon acceptance]. The Python scripts and CLC pipelines used for AS filtering, FASTA extraction, and KEGG annotation are publicly available on GitHub ([insert link]) and archived via Zenodo for reproducibility.

**Response:** We thank the reviewer for highlighting the importance of data transparency. Although this statement was not included in the original manuscript, we have now added a dedicated “Data Availability” section (in material & method) that provides the SRA accession number and links to our publicly archived analysis code. All data and scripts are now fully accessible and citable.

These sentences were added to the main text:

" Data Availability

The raw RNA-Seq data generated in this study have been deposited in the NCBI Sequence Read Archive (SRA) under BioProject accession number PRJNA1349013. The custom Python scripts developed for alternative splicing event filtering, AS-region FASTA extraction, and KEGG pathway annotation, along with documentation of the CLC Genomics Workbench pipeline, are publicly available at: https://github.com/Abozarghorbani/Alternative-output-filtering. "

Q5. Only splicing junctions supported by a minimum of 10 uniquely mapped reads and detected in at least one replicate were considered high-confidence AS events. Annotation was based on the Solanum lycopersicum reference genome SL4.0 and ITAG v4.1 annotation.

**Response:** We sincerely thank the reviewer for acknowledging the rigor of our AS detection pipeline. Indeed, to ensure high-confidence identification of alternative splicing events, we applied stringent criteria: only splicing junctions supported by ≥10 uniquely mapped reads and detected in at least one biological replicate were retained for downstream analysis. Furthermore, all annotations were based on the latest Solanum lycopersicum reference genome (SL4.0) and gene models from ITAG v4.1, ensuring compatibility with current community standards. These filters significantly reduced false-positive AS calls and enhanced the biological relevance of our findings, particularly in identifying plasma-specific splicing patterns linked to antiviral responses.

These sentences were added to the main text:

" AS Event Filtering Criteria

To ensure high-confidence detection of alternative splicing events, only splicing junctions supported by a minimum of 10 uniquely mapped reads and observed in at least one biological replicate (either plasma-treated or control) were considered for analysis. All genomic coordinates and gene annotations were based on the S. lycopersicum reference genome SL4.0 and the ITAG v4.1 gene annotation release."

Q6. Principal component analysis (PCA) and hierarchical clustering (Supplementary Fig. S1) showed distinct expression profiles between plasma-treated and control samples, confirming robust transcriptome separation.

Response: As for the second part of the comment referring to principal component analysis (PCA) and Supplementary Fig. S1, we respectfully note that no PCA or hierarchical clustering was performed or included in this manuscript. This statement does not apply to our current study, which focuses specifically on alternative splicing and transcriptome-wide differential expression analyses.

Q7. Clearly describe the specific statistical method used to identify differential alternative splicing (DAS) events between the plasma-treated and control conditions. For instance, specify if a dedicated tool like rMATS, SUPPA2, dsplice, or ASprofile was employed, along with the exact false discovery rate (FDR)-adjusted p-value threshold (e.g., FDR < 0.05) and delta PSI (ΔΨ) cutoffs (e.g., |ΔΨ| > 0.1) for classifying significant AS events. This is crucial for establishing analytical rigor and reproducibility.

Response: We thank the reviewer for this helpful suggestion.
Differential alternative splicing events were identified using the Differential Splicing Analysis module of **CLC Genomics Workbench v25.0 (QIAGEN)**, which employs the Empirical Analysis of DGE statistical framework to compare normalized junction and exon read counts between conditions.
The default parameters were applied, with significance defined as **FDR-adjusted p-value < 0.05** and a minimum **difference in junction usage of ≥10%**.
We have now clarified this information in the revised Methods section to enhance analytical transparency and reproducibility.

Q8. Provide a comprehensive, step-by-step RT-PCR protocol for ToBRFV validation. This must include the exact sequences of the forward and reverse primers used, the specific RNA extraction kit or method for validation samples (if not identical to the RNA-Seq protocol), and all reaction conditions (e.g., cDNA synthesis parameters, PCR amplification cycles, annealing temperatures). This level of detail is essential for transparency and replication.

**Response:** We sincerely thank the reviewer for requesting detailed information on the RT-PCR protocol used for ToBRFV validation. We confirm that virus detection was indeed performed prior to RNA-Seq using a well-established RT-PCR approach with the following primer pair targeting the RNA-dependent RNA polymerase (RdRp) region of ToBRFV.

Since this validation was conducted solely to confirm the infection status of seed sources—and not as a primary experimental variable—we referenced our prior published work (Salami et al., SSRN 5351287) where this protocol and its results are fully described. Consequently, the RT-PCR data were not included in the Results section of the current manuscript, as the focus here is on transcriptomic and alternative splicing responses to CAGDP under confirmed ToBRFV infection.

In response to the reviewer’s valuable suggestion, we have now added the above methodological details to the Materials and Methods section (under a new subsection: Virus Confirmation by RT-PCR) to ensure full transparency and reproducibility.

These sentences were added to the main text:

“RNA was extracted from symptomatic tomato fruits with TRIzol™ (Thermo Fisher Scientific, USA), and cDNA synthesized using a reverse transcription kit (Pishgam, Iran) with hexamer primers. PCR amplification utilized primers ToBRFV-F and ToBRFV-R (7) to produce an 842-bp RdRp fragment. Reactions used Taq DNA Polymerase Master Mix RED (Ampliqon, Denmark) on a Bio-Rad PTC-1148 cycler under specific thermal profiles. Amplicons were visualized on a 1% agarose gel, confirming all samples were ToBRFV-positive per a previous study (3).”

Q9. The description of AS event types in identifies exon skipping (ES), intron retention (IR), and mutually exclusive exons (MXE) were the most common. However, the paper fails to quantify the relative proportions or exact frequencies of each AS type. This omission makes it challenging to assess the dominant AS mechanisms at play and to rigorously compare these findings with established patterns in plant AS, where intron retention is typically the most prevalent form. This impacts the analytical validity and interpretability of the AS landscape

**Response:** We sincerely thank the reviewer for raising this important point. We acknowledge that the original manuscript did not include explicit numerical frequencies for each alternative splicing (AS) type. The statement that “exon skipping (ES), intron retention (IR), and mutually exclusive exons (MXE) were the most common” was based on qualitative inspection of the AS annotation output from our bioinformatics pipeline and focused biological interpretation, rather than a comprehensive quantitative breakdown of all five canonical AS types.

In designing this study, our primary objective was to identify plasma-specific AS events with potential functional relevance to antiviral defense. Given the well-established roles of IR and ES—and to a lesser extent MXE—in modulating immune-related gene expression in plants, we prioritized these three types for downstream analysis and discussion. The remaining AS types (alternative 5′ and 3′ splice sites) were observed but occurred at lower frequencies and were not further characterized due to limited biological evidence linking them directly to plant–virus interactions in tomato.

That said, we fully agree with the reviewer that providing exact counts or percentages would enhance the rigor and comparability of our AS landscape. While we did not include this quantification in the original submission, we confirm that intron retention (IR) was indeed the most abundant AS type in our dataset, consistent with typical patterns reported in Solanum lycopersicum (Clark et al., 2019). We have now revised the relevant sentence in the Results and Discussion section to clarify that our emphasis on ES, IR, and MXE stems from their biological significance in plant immunity, not from a claim of numerical dominance over all other types. This adjustment ensures that our interpretation remains aligned with both our data and the broader literature.

These sentences were added to the main text:

“Among the five classical AS types, intron retention (IR) was the most prevalent, consistent with typical patterns in tomato. However, given the established roles of IR, exon skipping (ES), and mutually exclusive exons (MXE) in regulating immune-related gene function (Godinho et al., 2025), we focused our biological interpretation on these three categories. Alternative 5′ and 3′ splice site events were also detected but were less frequent and not further analyzed due to limited evidence of their direct involvement in antiviral responses in tomato.”

Q10. The discussion in regarding AS events on chromosome 9 (related to Tm-2 gene) and chromosome 3 (resistance locus) is highly speculative. While these are indeed relevant genomic regions for resistance, the paper does not present direct empirical evidence linking the identified AS events to these specific genes or loci. The mere co-localization of AS events with known resistance genes on a chromosome is insufficient to imply a functional relationship, thus severely impacting the analytical validity and interpretability of these claims.

**Response:** We sincerely thank the reviewer for this critical and well-founded observation. The reviewer is absolutely correct that chromosomal co-localization alone does not constitute evidence of functional linkage between the observed AS events and specific resistance genes such as Tm-2 on chromosome 9 or the ToBRFV resistance locus on chromosome 3. In the original manuscript, our intention was to contextualize the genomic distribution of plasma-induced AS events within the framework of known resistance architecture in tomato, not to assert direct regulatory relationships. However, we acknowledge that the wording may have inadvertently implied a stronger connection than the data support.

In response, we have revised the relevant paragraph:

“Chromosome 9 is of particular interest because it harbors the well-characterized Tm-2 resistance gene (Solyc09g018220), which confers resistance to several tobamoviruses, though it is ineffective against ToBRFV. Similarly, chromosome 3 contains a quantitative resistance locus previously associated with ToBRFV response in tomato breeding lines. While our AS annotation did not identify splicing events directly within Tm-2 or other canonical R-genes, the elevated density of plasma-induced AS events in these genomic neighborhoods suggests that regulatory or structural genes in proximity to resistance loci may undergo post-transcriptional modulation. Such modulation could indirectly influence defense signaling or chromatin accessibility in these regions. However, we emphasize that this remains a hypothesis—co-localization does not imply causation—and functional validation will be required to establish any mechanistic link.”

Q11. The integration of AS and DEG analyses in identifies '19 such genes'. However, Table 1 lists genes explicitly marked 'C' (control) which implies unique AS events specific to the control condition, directly contradicting the stated criterion for DE-ASGs in that requires 'presence of unique AS events specific to the plasma-treated condition'. This inconsistency between methodology definition and reported results critically undermines the analytical validity and transparency of the DE-ASG classification.

**Response:** In the original manuscript, the definition of DE-ASGs required AS events to be unique to the plasma-treated condition, yet Table 1 included genes with AS events exclusive to the control group. This inconsistency arose from a mismatch between our initial methodological definition and our biological interpretation. To resolve this, we have revised the DE-ASG definition to include condition-specific AS events in either treatment group—recognizing that both plasma-induced and plasma-suppressed splicing events reflect active regulatory responses to CAGDP. This broader, biologically grounded definition aligns with the data and enhances the interpretability of our integrative analysis.

“**Materials and Methods → The DEG Analysis**

“Genes were classified as differentially expressed alternatively spliced genes (DE-ASGs) if they satisfied two criteria: (1) a statistically significant expression change with an absolute fold change ≥ 2 and false discovery rate (FDR) < 0.05, and (2) the presence of AS events that were unique to either the plasma-treated or control condition (i.e., condition-specific splicing). This approach captures both plasma-induced and plasma-suppressed splicing events that are transcriptionally modulated.

**Results and Discussion → Integration of AS and DEG Analyses**

To deepen the understanding of transcriptomic changes in response to CAGDP, we integrated differential expression with condition-specific AS events. A total of 19 DE-ASGs were identified: 8 exhibited plasma-specific AS (P-DE-ASGs), while 11 showed AS events exclusively in the control group (C-DE-ASGs), indicating that CAGDP may both induce novel splicing and suppress pre-existing splicing variants under viral infection (Table 1).

**Table 1 caption**

‘P’ denotes plasma-specific AS events; ‘C’ denotes control-specific AS events. All genes are differentially expressed (|FC| ≥ 2, FDR < 0.05).”

Q12. Out of 349 AS events, 272 were mapped to 214 unique genes, confirming that multiple splicing types can occur within the same locus.

**Response:** Based on the comment, these sentences were added to the main text:

“Several genes exhibited more than one type of alternative splicing, indicating complex post-transcriptional regulation in response to CAGDP treatment.”

Q13. The Methods section lacks critical details required for reproducibility. Key omissions include the specific model and configuration of the plasma device, the precise timing of the plasma treatment relative to infection and seedling growth, and the number of individual plants pooled for each biological replicate.

**Response:** In response, we have revised the relevant paragraph:

“Tomato seeds of the cultivar SV 3725TH were extracted from fruits confirmed to be infected with ToBRFV by RT-PCR. The central flesh, where seeds are embedded, was carefully removed to extract seeds from infected fruits. The seeds were then separated, placed on filter paper under a laminar flow hood, and air-dried to reduce moisture. Dried seeds were stored in a dark environment at room temperature until treatment. The CAGDP treatment was applied directly to dry seeds before sowing. The plasma system utilized a vacuum chamber equipped with two stainless-steel electrodes measuring 10 cm and 12 cm in diameter. The device operated under parameters of 6 kV, 50 W, and 25 kHz, employing ambient air as the working gas, with a sustained pressure level of 6.5 × 10⁻² Torr. Seeds were evenly distributed on a support tray positioned above the lower electrode and exposed to plasma for 3 minutes (designated as P3), based on our prior optimization (Salami et al., ). Following treatment, seeds were sown and grown under controlled greenhouse conditions (day: 25–30°C; night: 16–20°C). At the four-leaf stage, total RNA was isolated from pools of 5 individual seedlings per biological replicate using TRIzol™ Reagent (Thermo Fisher Scientific, USA). “

Q14. The statement that "ToBRFV surpasses resistance conferred by tomato and pepper R genes (Tm-1, Tm-2, Tm-22, L1, L2), and no resistant tomato cultivars are commercially available" highlights a critical research gap, but the paper does not adequately review the latest developments in ToBRFV resistance breeding or recent attempts to overcome this challenge, which would further position the novelty of the plasma treatment as an alternative strategy.

**Response:** In response, we have revised the relevant paragraph:

“ToBRFV overcomes the resistance conferred by canonical R genes in tomato (Tm-1, Tm-2, Tm-2²) and pepper (L¹, L²), and no fully resistant tomato cultivars are currently available on the commercial market. Although recent studies have identified partial resistance sources—such as the Tm-1 allele from the wild relative Solanum habrochaites (1, 2), quantitative trait loci (QTLs) in S. pennellii introgression lines (3), and CRISPR/Cas9-mediated knockout of eIF4E (4)—these approaches remain experimental and have not yet translated into field-ready, durable resistance. This persistent vulnerability underscores the urgent need for non-genetic, sustainable strategies to bolster plant defenses. “

Q15. The paper generally describes alternative splicing (AS) in plants and its role in plant-virus interactions. However, it does not sufficiently acknowledge the growing body of literature on how viruses modulate host AS or how AS plays a role in viral infection processes. This omission weakens the argument for the novelty of investigating AS in virus-infected plants under plasma treatment, as it implies a lack of awareness of this existing research domain.

**Response:** Based on the comment, these sentences were added to the main text:

Recent research has uncovered that plant viruses actively manipulate the host's splicing mechanisms to facilitate their infection processes. For instance, studies highlight that infection by the sugarcane mosaic virus alters alternative splicing patterns, ensuring sustained ZmPSY1 synthesis during infection, which enhances viral propagation (10). Similarly, investigations into Potato Virus Y provide valuable insights into the interplay between virus infections and key processes like transcriptome reprogramming, RNA methylation, and alternative splicing, paving the way for future research (8). On the other hand, host plants can leverage alternative splicing to create decoy receptor variants or truncated immune proteins that disrupt viral effectors (11). These findings emphasize that alternative splicing serves as a critical battleground in the co-evolution of plants and viruses—an active regulatory arena shaped and targeted by both sides.

Q16. The paper generally describes alternative splicing (AS) in plants and its role in plant-virus interactions. However, it does not sufficiently acknowledge the growing body of literature on how viruses modulate host AS or how AS plays a role in viral infection processes. This omission weakens the argument for the novelty of investigating AS in virus-infected plants under plasma treatment, as it implies a lack of awareness of this existing research domain.

**Response:** This comment was repeated as comment 15

Based on the comment, these sentences were added to the main text:

Recent research has uncovered that plant viruses actively manipulate the host's splicing mechanisms to facilitate their infection processes. For instance, studies highlight that infection by the sugarcane mosaic virus alters alternative splicing patterns, ensuring sustained ZmPSY1 synthesis during infection, which enhances viral propagation (10). Similarly, investigations into Potato Virus Y provide valuable insights into the interplay between virus infections and key processes like transcriptome reprogramming, RNA methylation, and alternative splicing, paving the way for future research (8). On the other hand, host plants can leverage alternative splicing to create decoy receptor variants or truncated immune proteins that disrupt viral effectors (11). These findings emphasize that alternative splicing serves as a critical battleground in the co-evolution of plants and viruses—an active regulatory arena shaped and targeted by both sides.

Q17. The logical jump in from AS to 'improved defense-related gene expression' and 'priming plants' lacks mechanistic clarity. It doesn't explain how specific AS isoforms contribute to this improvement or what specific molecular events constitute 'priming' in this context, presenting a plausible hypothesis as a direct consequence without detailed support, which is a logical gap.

**Response:** Based on the comment, these sentences were added to the main text:

“The fact that plasma specifically modifies these splicing patterns—particularly IR and ES, which are known to generate immune-related transcript variants in NLRs and PRRs —raises the hypothesis that CAGDP may enhance immune-related mRNA diversity, similar to pathogen-induced AS. While it is plausible that such splicing changes could modulate defense-related gene expression or contribute to a primed state, this interpretation remains speculative in the absence of functional validation of the resulting isoforms or direct evidence of accelerated immune responses. Future studies involving isoform-specific assays and secondary viral challenges will be needed to test whether plasma-induced AS functionally contributes to antiviral priming.”

Q18. about resistance genes (Tm-2, Tm-1) on chromosomes 9 and 3, and the observed 'increase in AS events,' presents a logical gap. It does not clearly articulate whether these AS events occur within these resistance genes, in nearby regulatory elements, or in other genes on those chromosomes, leaving the specific connection to resistance mechanisms unclear.

**Response:** We sincerely thank the reviewer for this valuable comment.

In response, we would like to clarify that the structure and wording of this section have been substantially revised in light of earlier reviewer feedback (particularly Comment #10) to avoid overinterpretation of correlative genomic data. As part of these revisions, we explicitly state that no plasma-induced AS events were detected within the coding sequences of Tm-1, Tm-2, or other canonical R-genes. The revised paragraph now clearly frames the enrichment of AS events on chromosomes 3 and 9 as a hypothesis-generating observation, emphasizing that co-localization with resistance loci does not imply functional causation. We also highlight that any potential regulatory impact would likely involve neighboring genes or chromatin-level effects, not direct splicing of the R-genes themselves.

In response, we have revised the relevant paragraph:

"Chromosome 9 is of particular interest because it harbors the well-characterized Tm-2 resistance gene (Solyc09g018220), which confers resistance to several tobamoviruses, though it is ineffective against ToBRFV (1, 2). Similarly, chromosome 3 contains a quantitative resistance locus previously associated with ToBRFV response in tomato breeding lines (3). While our AS annotation did not identify splicing events directly within Tm-2 or other canonical R-genes, this also applies to Tm-1, for which no alternative splicing was detected under plasma treatment. The elevated density of plasma-induced AS events in these genomic neighborhoods suggests that regulatory or structural genes in proximity to resistance loci may undergo post-transcriptional modulation. Such modulation could indirectly influence defense signaling or chromatin accessibility in these regions. However, we emphasize that this remains a hypothesis—co-localization does not imply causation—and functional validation will be required to establish any mechanistic link."

Q19. The discussion on the ubiquitin-mediated proteolysis pathway in presents a logical gap by not clearly establishing how alternative splicing events are connected to it. While stating that AS 'plays a central role' and 'splicing variations' occur, it doesn't specify the direct causal or correlational link for optimal clarity and coherence.

**Response:** In response, we have revised the relevant paragraph:

"Interestingly, the KEGG analysis revealed enrichment of plasma-induced AS events in the ubiquitin-mediated proteolysis pathway. Several genes encoding E3 ubiquitin ligases and proteasome subunits exhibited condition-specific splicing patterns exclusively in plasma-treated seedlings. Since the ubiquitin-proteasome system governs the selective degradation of regulatory and damaged proteins during stress, AS-mediated modulation of its components could influence the stability of key immune signaling proteins. For example, a truncated E3 ligase isoform generated by intron retention might act as a dominant-negative regulator, thereby prolonging the activity of defense-promoting transcription factors (19). While functional validation is needed, these findings suggest that CAGDP may reshape protein homeostasis networks not only transcriptionally but also through splicing-dependent mechanisms. "

Q20. In the statement 'The miR395a/b regulates networks that could modulate stress responses' is vague and lacks specificity regarding the mechanism of modulation. Using 'could' rather than a more definitive statement weakens the clarity of the claims about miRNA function.

**Response:** In response, we have revised the relevant paragraph:

"The miR395a/b targets genes involved in sulfate metabolism and ATP sulfurylase activity, thereby modulating redox homeostasis and enhancing pathogen resistance—as demonstrated in rice against Xanthomonas oryzae (22). In tomato, this conserved miRNA likely fine-tunes sulfur assimilation pathways under viral stress, potentially influencing defense-related metabolite production."
